# Spatiotemporal Relationship of Brain Pathways during Human Fetal Development Using High-Angular Resolution Diffusion MR Imaging and Histology

**DOI:** 10.3389/fnins.2017.00348

**Published:** 2017-07-11

**Authors:** Lana Vasung, Marina Raguz, Ivica Kostovic, Emi Takahashi

**Affiliations:** ^1^Division of Newborn Medicine, Boston Children's Hospital, Harvard Medical School Boston, MA, United States; ^2^School of Medicine, Croatian Institute for Brain Research, University of Zagreb Zagreb, Croatia

**Keywords:** fiber tractography, human fetal brain, gyrification, histology, comparative, axonal development

## Abstract

In this study, we aimed to identify major fiber pathways and their spatiotemporal relationships within transient fetal zones in the human fetal brain by comparing postmortem high-angular resolution diffusion MR imaging (HARDI) in combination with deterministic streamline tractography and histology. Diffusion weighted imaging was performed on postmortem human fetal brains [*N* = 9, age = 18–34 post-conceptual weeks (PCW)] that were grossly normal with no pathologic abnormalities. After HARDI was performed, the fibers were reconstructed using Q-ball algorithm and deterministic streamline tractography. The position of major fiber pathways within transient fetal zones was identified both on diffusion weighted images and on histological sections. Our major findings include: (1) the development of massive projection fibers by 18 PCW, as compared to most association fibers (with the exception of limbic fibers) which have only begun to emerge, (2) the characteristic laminar distribution and sagittal plane geometry of reconstructed fibers throughout development, (3) the protracted prenatal development shown of the corpus collosum and its' associated fibers, as well as the association fibers, and (4) the predomination of radial coherence in the telencephalon (i.e., majority of streamlines in the telencephalic wall were radially oriented) during early prenatal period (24 PCW). In conclusion, correlation between histology and HARDI (in combination with Q-ball reconstruction and deterministic streamline tractography) allowed us to detect sequential development of fiber systems (projection, callosal, and association), their spatial relations with transient fetal zones, and their geometric properties.

## Introduction

The development of fiber pathways in the human brain is a prolonged process that starts during the embryonic period (His, 1904; Hochstetter, 1919; Kostović, 1991; Dubois et al., 2015) and lasts until young adulthood (i.e., the final phase of myelination; Flechsig, 1920; Yakovlev and Lecours, 1967; Brody et al., 1987; Kinney et al., 1988). The majority of fiber pathways in humans are also present in non-human primates, and can be identified and analyzed in detail by fiber tracing methods (Schmahmann and Pandya, 1992, 2009). However, in humans, the study of fiber pathways is limited by quantity and quality of available postmortem materials (Von Monakow, 1905), and can only be grossly identified using the Klingler's method that is based on fiber dissection of the brain (Ludwig and Klingler, 1956; Klingler and Gloor, 1960; Zemmoura et al., 2016), or based on secondary axonal degeneration (Von Monakow, 1905; Schmahmann and Pandya, 2009). Nevertheless, compared to the study of the adult human brain, studying the development of fibers using histology during fetal and perinatal periods may be slightly easier. This is mostly because, during these periods, different groups of fibers sequentially emerge, show simpler and shorter trajectories, and undergo myelination (Brody et al., 1987; Kinney et al., 1988). On top of these reasons, it also might be easier to study the development of the fibers in the fetal brain due to changes in their chemical properties (Kostovic and Goldman-Rakic, 1983; Vasung et al., 2010). Nonetheless, it is difficult to analyze detailed 3-dimensional structures of fiber pathways in human brains solely using histology techniques.

Diffusion MRI (dMRI) is an imaging method that relies on diffusion of water molecules. Fiber tractography, based on dMRI, has a unique ability to detect, both *in vivo* and *in vitro*, the three dimensional fiber architecture of the human brain. Although, these techniques have gained enormous popularity over the past decade, they still suffer from significant limitations (e.g., Jones et al., 2013). Currently available diffusion models are numerous [e.g., DTI (Basser et al., 1994); CHARMED (Assaf and Basser, 2005), diffusion orientation transform (Özarslan et al., 2006), and spherical deconvolution (Tournier et al., 2004)]. In addition, equally numerous numbers of tractography algorithms, both deterministic and probabilistic, are also available [e.g., streamline (Mori et al., 1999), tensor deflection algorithms (Weinstein et al., 1999), q-ball tracking (Chao et al., 2007), multi-tensor tracking (Kreher et al., 2005), and diffusion spectrum imaging (DSI; Tuch, 2002)]. Regardless of the high number of available tractography and diffusion algorithms, the most suitable tractography algorithm for specific subjects or specimens in a research study is still difficult to choose due to the lack of the ground truth (e.g., histological confirmation of anatomical fiber architecture). Finally, there are also several dMRI analysis tools that were recently developed in order to visualize complexity of connections in the white matter (Sporns et al., 2005; Hagmann et al., 2008; Iturria-Medina et al., 2008; Van Den Heuvel and Sporns, 2011). However, a recent study suggested that currently available tools for dMRI analyses of structural connectivity are potentially dominated by false fiber pathways (Maier-Hein et al., 2016). Therefore, the known limitations of dMRI, such as current possible spatial resolution that makes it difficult to determine more detailed angular information as well as to detect laminar and columnar origins of tractography pathways [for details, see (Jones et al., 2013)], lead to challenges in the evaluation of functional significance of specific fiber pathways.

When histologically staining fiber pathways during prenatal developmental periods, it is possible to study their maturation as chemical changes occur gradually from their origin to their terminal target (Kostović, 1991; Eyre et al., 2000; Vasung et al., 2010). Although, diffusion MRI tractography has proven to be successful in the study of prenatal fiber pathways in postmortem specimens (Takahashi et al., 2012; Huang et al., 2013; Xu et al., 2014) and *in vivo* subjects (Kasprian et al., 2008; Kim et al., 2008; Zanin et al., 2011), due to its known limitations, histological knowledge on axonal growth is very important and useful as a reference. Existing diffusion MRI data on prenatal development of fiber pathways showed good correlation with histological data (Vasung et al., 2010; Takahashi et al., 2012). However, the knowledge of spatiotemporal and sequential development of major fiber pathways still remains fragmented. In this study, we take advantage of high-angular resolution diffusion MRI (HARDI) acquisition and Q-ball reconstruction of fiber orientations using the spherical harmonic basis in combination with a deterministic streamline tractography algorithm (Hess et al., 2006) to analyze the growth of projection, commissural, and long cortico-cortical association pathways during mid-fetal and prenatal periods. These periods are crucial for the study of e.g., hypoxic ischemic lesions of white matter, the most common neurological injury in preterm infants (Volpe, 2009). In addition, we hope to find normative data to study fine abnormalities of connectivity leading to a large array of neurodevelopmental disorders.

## Materials and methods

### Materials

In total, nine postmortem human fetal brains {*N* = 7 mid-fetal period [18–23 postconceptional weeks (PCW)], *N* = 1 [early preterm (23–28 PCW)], and *N* = 1 [late preterm (29–34 PCW)]} were imaged. Postmortem specimens were obtained with full parental consent, through the Department of Pathology, Brigham and Women's Hospital (BWH; *N* = 6) and the Allen Institute Brain Bank (*N* = 3), under protocols approved by each institutional review board for human research. The primary cause of death was complications of prematurity. All the brains were grossly normal with no pathologic abnormalities identified at the macroscopic level, or during routine autopsy examination. Images of the histology sections were obtained from Croatian Institute for Brain Research (Judas et al., 2011) and from Brigham and Women's Hospital. Each institutional review board for human research approved the protocols for histology.

### MRI procedure

The brains were fixed in 4% paraformaldehyde and were placed in Fomblin solution during scan. They were imaged with a 4.7 T Bruker Biospec MR using following sequence: 3D diffusion-weighted spin-echo echo-planar imaging (TR/TE = 1,000/40 ms); with an imaging matrix of 112 × 112 × 112 pixels; 60 diffusion-weighted measurements (*b* = 8,000 s/mm^2^) and one non-diffusion-weighted measurement (*b* = 0 s/mm^2^); spatial resolution = 440 × 500 × 500 μm. The detail description of the postmortem imaging protocol can be found in our past studies (Takahashi et al., 2012).

### Tissue segmentation and fiber pathway reconstruction

Following the anatomical landmarks previously described (Vasung et al., 2016), the DWI and FA images of the fetal brains were manually segmented into following regions; background, cortical plate, proliferative zones (ventricular and subventricular zone), subplate zone, thalamus, basal ganglia, pons, and mesencephalon (see Supplementary Figure 1). These regions were used as regions of interest (ROIs) for further analyses. Images were segmented using the ITK snap software (www.itksnap.org).

In this study, we used HARDI acquisition methods followed by Q-ball reconstruction of multiple fiber orientations using the spherical harmonic basis (for details, see Hess et al., 2006). With this approach we were able to detect multiple local maxima on an orientation distribution function (ODF). Using each pair of local maxima on an ODF, we applied the streamline algorithm (e.g., Mori et al., 1999) using DiffusionToolkit (trackvis.org) to initiate and continue tractography (Tuch et al., 2003) in order to identify crossing pathways within single voxels. The streamline trajectories were reconstructed by continuously pursuing the orientation vector of least curvature (Conturo et al., 1999). The term “streamline” refers to connecting tractography pathways using a local maximum or maxima, which can be applied to both DTI and HARDI. The streamline technique is limited in its ability to resolve crossing pathways when used with the traditional DTI technique as tractography pathways are established by connecting the direction of the principal eigenvector of a tensor. The number of seeds per voxel was 1, however, we did produce more streamlines than 1 when ODFs indicated multiple directions of water diffusivity. The reconstructed streamlines were terminated if the orientation vector angle was >40°, or if those fiber pathways extended outside of the pial surface region of interest. We did not use FA threshold to terminate fiber tracking as areas with ongoing myelination (Flechsig, 1920; Brody et al., 1987; Kinney et al., 1988) and fiber crossings (Judas et al., 2005) in the developing brain tend to have lower FA values. These low FA regions could lead to false termination of fiber pathways (Takahashi et al., 2010). In addition, it is worth mentioning that nowadays in the adult brain, tractography is mostly performed using a WM/GM mask, since FA threshold can cause similar problems (for details see Smith et al., 2012; Chamberland et al., 2014; Girard et al., 2014).

### Segmentation of fiber pathways

Depending on the region and age, the fiber pathways were segmented using the one- or two-ROI approach (Catani and Thiebaut De Schotten, 2008). One-ROI approach was employed in order to reconstruct tracts with known anatomical landmarks, while two-ROI approach was employed in order to identify the end/origin points of certain fibers (callosal) or specific streamlines (radial fibers). After reconstructing specific tracts using the predefined anatomical knowledge, we excluded all the streamlines that do not anatomically correspond to the specific tract. These streamlines were excluded by creating additional ROIs and by choosing a NO PART operator. In short, using the segmented ROIs, the following sets of fibers were segmented:

#### Projection

- thalamocortical fibers (one-ROI approach from thalamus),
- tegmental fibers (one-ROI approach from mesencephalon),
- basal forebrain and striatal fibers conveyed to the external capsule (one-ROI approach from regions below putamen and the external capsule),
- fasciculus subcallosus—Muratoff bundle [one-ROI approach from ROI placed in the periventricular fiber rich zone described previously (Vasung et al., 2011)],
- corticopontine and fibers related to pons (one-ROI approach from pons),
- geniculocortical fibers (one-ROI approach from lateral geniculate body)

#### Association

- fronto-occipital fasciculus [one-ROI approach from ROI at the level (in central brain regions) and above the level (in frontal and occipital regions) of corticostriatal junction (Vasung et al., 2011)],
- inferior fronto-occipital fasciculus [one-ROI approach from ROI placed in the external/extreme capsule (Obersteiner and Redlich, 1902; Schmahmann and Pandya, 2009), fibers running in anterio-posterior direction were selected]

In order to identify long association fibers that are still growing, we have used a one-ROI approach where specific parts of cortical plate were used as ROIs. For example, in order to track the middle longitudinal fascicle, the cortical plate of the superior temporal gyrus was repainted and used as a new ROI; for segmentation of the vertical occipital fasciculus, the segmented cortical plate of the lateral occipital lobe was repainted and used as a new ROI; for the segmentation of inferior longitudinal fasciculus, the segmented cortical plate of the inferior temporal gyrus was repainted and used as a new ROI; for segmentation of the superior longitudinal fasciculus (subcomponent III), the segmented subplate zone was restricted to the regions of the inferior frontal gyrus, ventral parts of precentral and postcentral gyrus, and angular gyrus using the coronal, sagittal, and axial slices, the restricted subplate zone of these regions was used as a new ROI.

#### Callosal

Callosal fibers that originated/ended in the cortical plate were segmented using the two-ROI approach (segmented corpus callosum in the mid-sagittal plane and cortical plate). Callosal fibers that originated/ended in the subplate plate were segmented using the two-ROI approach (segmented corpus callosum in the mid-sagittal plane and subplate zone).

Lastly, we used a NOT operator for certain overlapping fibers in order to ensure segmentation of specific fiber bundles. E.g., when segmenting fasciculus subcallosus-Muratoff bundle we used the NOT operator to exclude all the fibers that pass through the ROI but do not anatomically correspond to origin/termination of the fibers of the fasciculus subcallosus (pons, external capsule, thalamus, tegmentum, medulla).

All the fibers were reconstructed in 3D, and were superimposed to the reconstructed 3D surfaces of similar aged fetal brains from Zagreb Neuroembryological Collection (for details on reconstruction of fetal surfaces see Vasung et al., 2016) using the Adobe Photoshop software. In order to ensure an illustration that is correct, we have firstly produced a rendered 3D volume of the cortical plate in TrackVis. After adjusting the opacity of the cortical plate, reconstructed fiber tracks were displayed and their relation with surface landmarks was visible (e.g., insula, temporal pole). Afterwards, we replaced the reconstructed volume rendered 3D cortical plate surface image with the high quality cortical plate surface images, preserving the spatial relations between surface landmarks and reconstructed fiber tracks.

#### Radial coherence

In order to distinguish different components of radially oriented streamlines, we have segmented several sets of radially oriented streamlines. The three radial oriented streamlines we selected for include: streamlines that stretched between cortical plate and proliferative zones (two-ROI approach, reconstruction of streamlines that connect cortical plate and proliferative zones), streamlines that originated in cortical plate but did not reach proliferative zone (one-ROI approach from cortical plate, operator NOT for proliferative zone ROI), and streamlines that originated in proliferative zones but did not reach the cortical plate (one-ROI approach from proliferative zones, operator NOT for cortical plate ROI).

### Comparison of fiber pathway trajectories with the histological sections

Reconstructed fiber pathway trajectories were superimposed on the corresponding FA images. In order to identify histological counterparts of regions that are occupied with segmented fiber pathways, we have used corresponding Nissl stained histological sections from Zagreb Neuroembryological Collection. For details of the collection and staining protocol please see (Judas et al., 2011). For the 18 PCW old specimen, hematoxylin and eosin-stained histological sections were obtained at Department of Pathology, Brigham and Women's Hospital.

## Results

### Mid-fetal period (18–23 PCW)

#### Projection fibers

**Figure 1:**
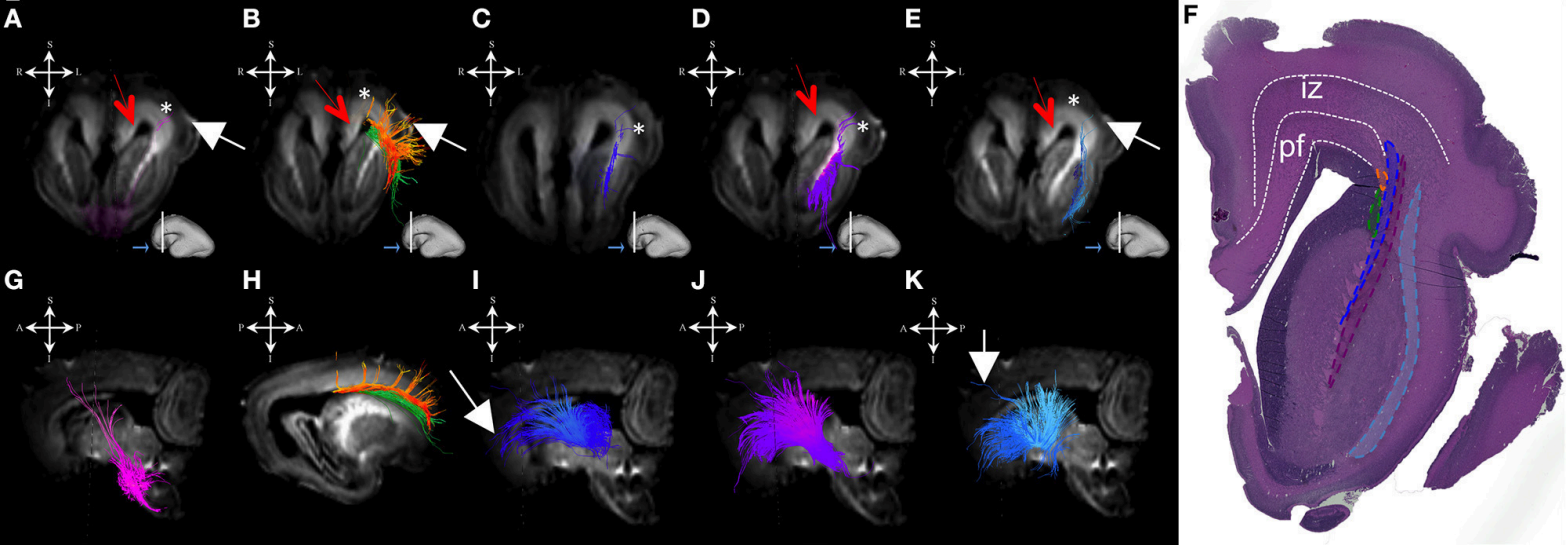
Projection pathways and fasciculus subcallosus during mid-fetal period. Reconstruction of projection corticopontine, ponto-cerebellar fibers, and fibers related to pons **(A,G)**, fasciculus subcallosus—Muratoff bundle (**B,H**, in green), thalamic **(C,I)**, tegmental **(D,J)**, and basal ganglia and basal forebrain fibers conveyed to external capsule **(E,K)** in 18 PCW old brain. Reconstruction of association fronto-occipital fasciculus (FOF) is shown in **(B,H)** orange. Coronal sections of DWI images are in the upper row, sagittal sections are in the bottom row. Adjacent to the each coronal slice is an illustration of the reference brain surface with the approximate level of the coronal slice (white line), and the view of the slice (anterior or posterior, marked with the blue arrow). Reference orientations [anterior (A), posterior (P), superior (S), inferior (I), left (L), and right (R)] are placed in the left upper corner of each slice. Subplate zone is marked with ^*^, cortical plate is marked with white arrow. Red arrows indicate periventricular fiber rich zone. **(F)** Haematoxylin and eosin stained coronal section of the similar aged brain (18 PCW). Regions that are occupied by specific fiber bundles found in **(A–E)** are encircled in histological section and marked by corresponding color (fronto-occipital fasciculus in orange, subcallosal fasciculus in green, thalamocortical fibers in blue, tegmental fibers in purple, and external capsule in light blue). The border between transient fetal zones is marked by dashed line. Abbreviations: iz (intermediate zone), pf (periventricular fiber rich zone).

**Figure 2:**
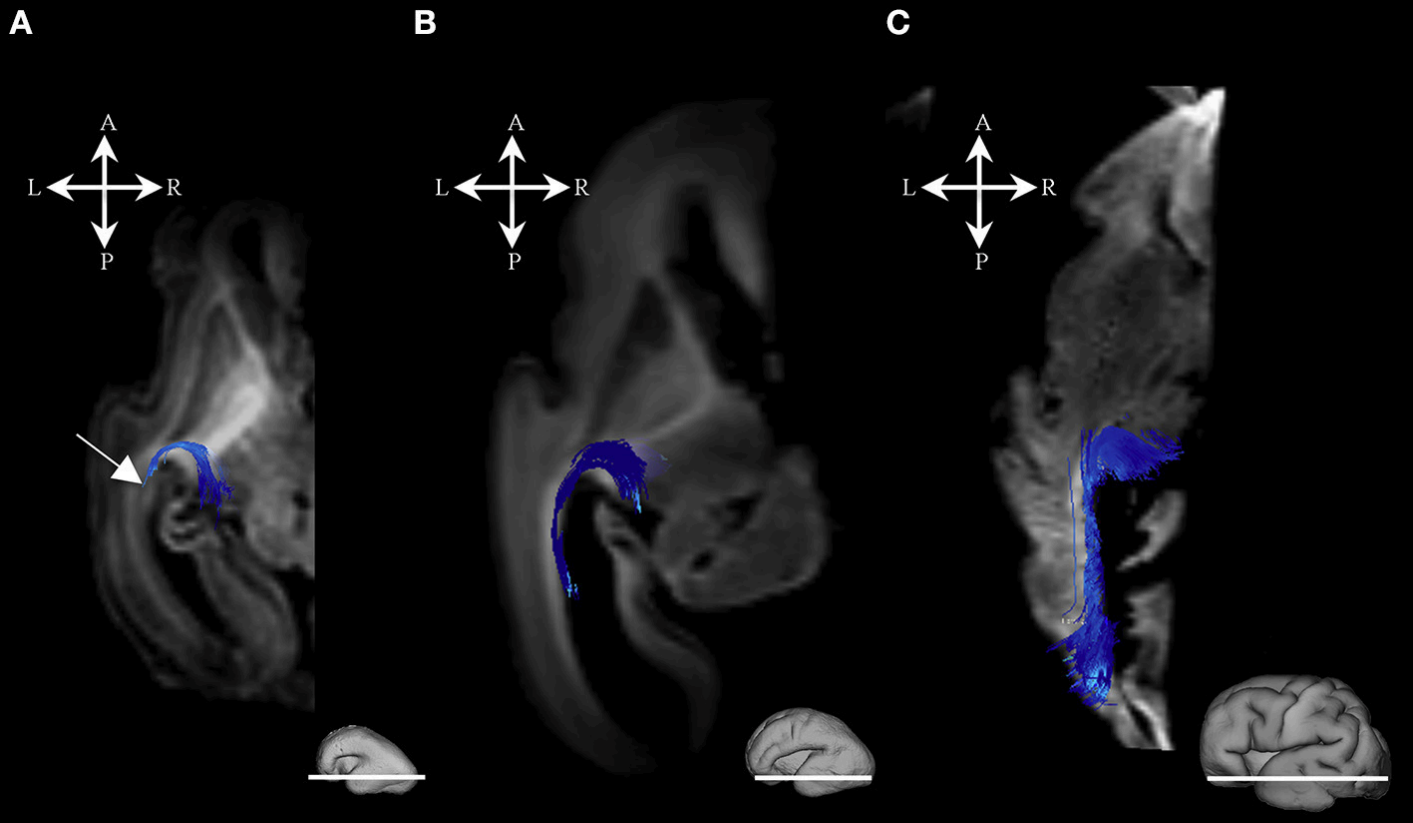
Geniculocortical fibers during mid-fetal and prenatal periods. Reconstruction of the geniculocortical fibers forming a loop in the 18 PCW **(A)**, 24 PCW **(B)**, and 30 PCW **(C)** old fetal brain. Reconstructed fibers are superimposed on the axial slices of corresponding DWI images. Adjacent to the each slice is an illustration of the reference brain surface with the approximate level of the axial slice (white line). Reference orientations [anterior (A), posterior (P), superior (S), inferior (I), left (L), and right (R)] are placed in the left upper corner of each slice. Note that the growing loop (corticopetal fibers) enters the sagittal strata (Figure 1, arrow). A part of loop resembles the Meyer's loop of the geniculocortical projection.

During this period, we have been able to identify a majority of the projection fiber pathways (Figure 1). The projection fiber pathways showed a medial-to-lateral arrangement (Figures 1A,E). By 18 PCW, the following fiber pathways were identified: cortico-ponto-cerebellar and fibers related to pons (Figures 1A,G pink), basal ganglia fibers that are conveyed to (i) fasciculus subcallosus-Muratoff bundle (Figures 1B,H green), and to (ii) the external capsule (Figures 1E,K light blue), tegmental fibers (Figures 1D,J purple), thalamic fibers (Figures 1C,I dark blue), and fibers from basal forebrain conveyed to the external capsule (Figures 1E,K light blue). During mid-fetal period, these fibers were occupying the periventricular fiber rich zone (cortico-pontine and fasciculus subcallosus) and intermediate zone (thalamic, tegmental, and basal ganglia and basal forebrain fibers conveyed to the external capsule). Their position was easily identified on Nissl stained section (Figure 1F). The majority of the projection fibers were connecting the subplate zone (Figure 1, asterisks) with subcortical structures, and a relatively few fibers were connecting the cortical plate (Figure 1 white arrows) with subcortical structures. During this phase, we have reconstructed geniculocortical fibers directed toward the developing temporal lobe, forming a loop which may correspond to a part of the geniculocortical projection, which forms a Meyers's loop and enters the sagittal stratum (Figure 2A white arrow). Nevertheless, these fibers did not reach the occipital cortical plate (Figure 2A).

#### Radial coherence

**Figure 3:**
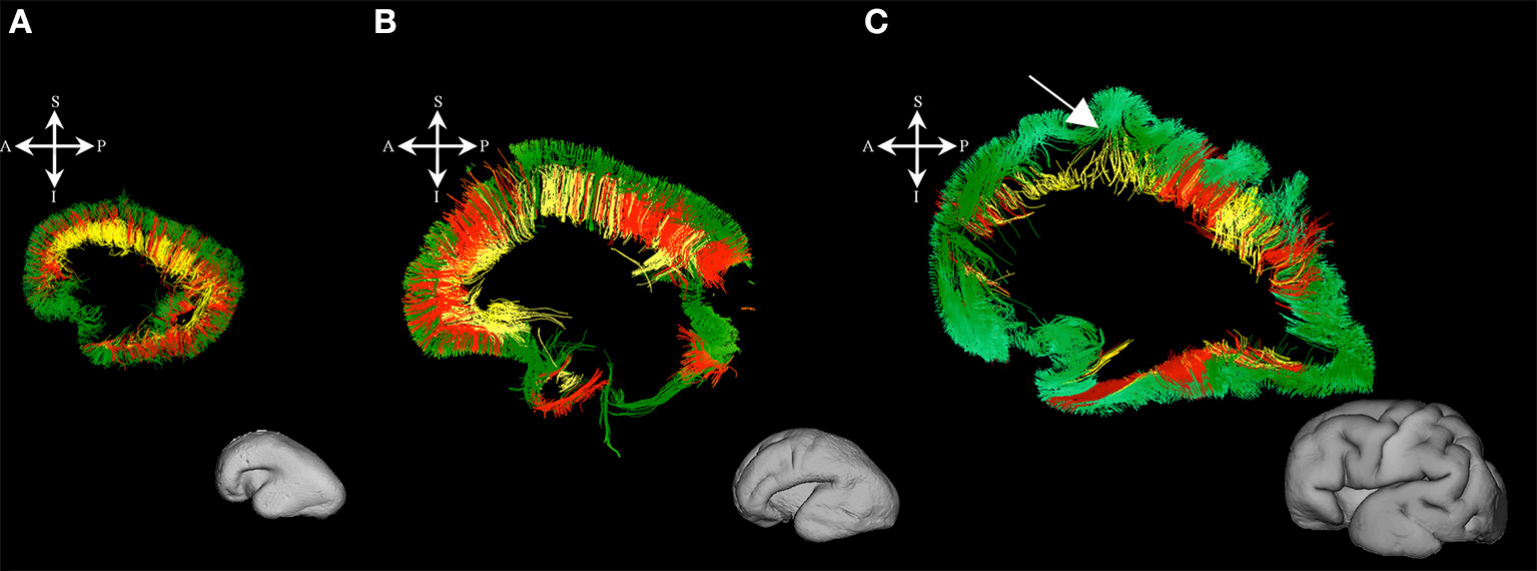
Radial coherence of the telencephalic wall during mid-fetal and prenatal periods. Reconstruction of radial streamlines stretching from cortical plate to the subplate/or intermedial zone (in green) in 18 PCW **(A)**, 24 PCW **(B)**, and 30 PCW **(C)** old fetal brains. Adjacent to the each sagittal slice is an illustration of the reference brain surface reconstructions of the 18 PCW **(A)**, 24 PCW **(B)**, and 30 PCW **(C)** brains of similar age from Zagreb Neuroembryological Collection. Reference orientations [anterior (A), posterior (P), superior (S), inferior (I), left (L), and right (R)] are placed in the left upper corner of each slice. Streamlines connecting proliferative zones with lower subplate zone (in yellow), streamlines connecting proliferative zones and cortical plate (in red). Arrow in C indicates fibers stretching from one gyrus to adjacent gyrus (corticocortical fibers) showing U-shape.

During this phase, we observed radial coherence (majority of streamlines starting/ending in cortical plate were radially oriented) of the telencephalic wall. Several sets of radial streamlines were reconstructed during this stage (Figure 3A). We reconstructed streamlines that were radially oriented and extended between proliferative zones (ventricular and subventricular zone) and the cortical plate (Figure 3A red), streamlines that stretched between proliferative zones (ventricular and subventricular zone) and the subplate without reaching the cortical plate (Figure 3A yellow), and streamlines that connected the cortical plate and the intermediate zone (Figure 3A green).

#### Callosal fibers

**Figure 4:**
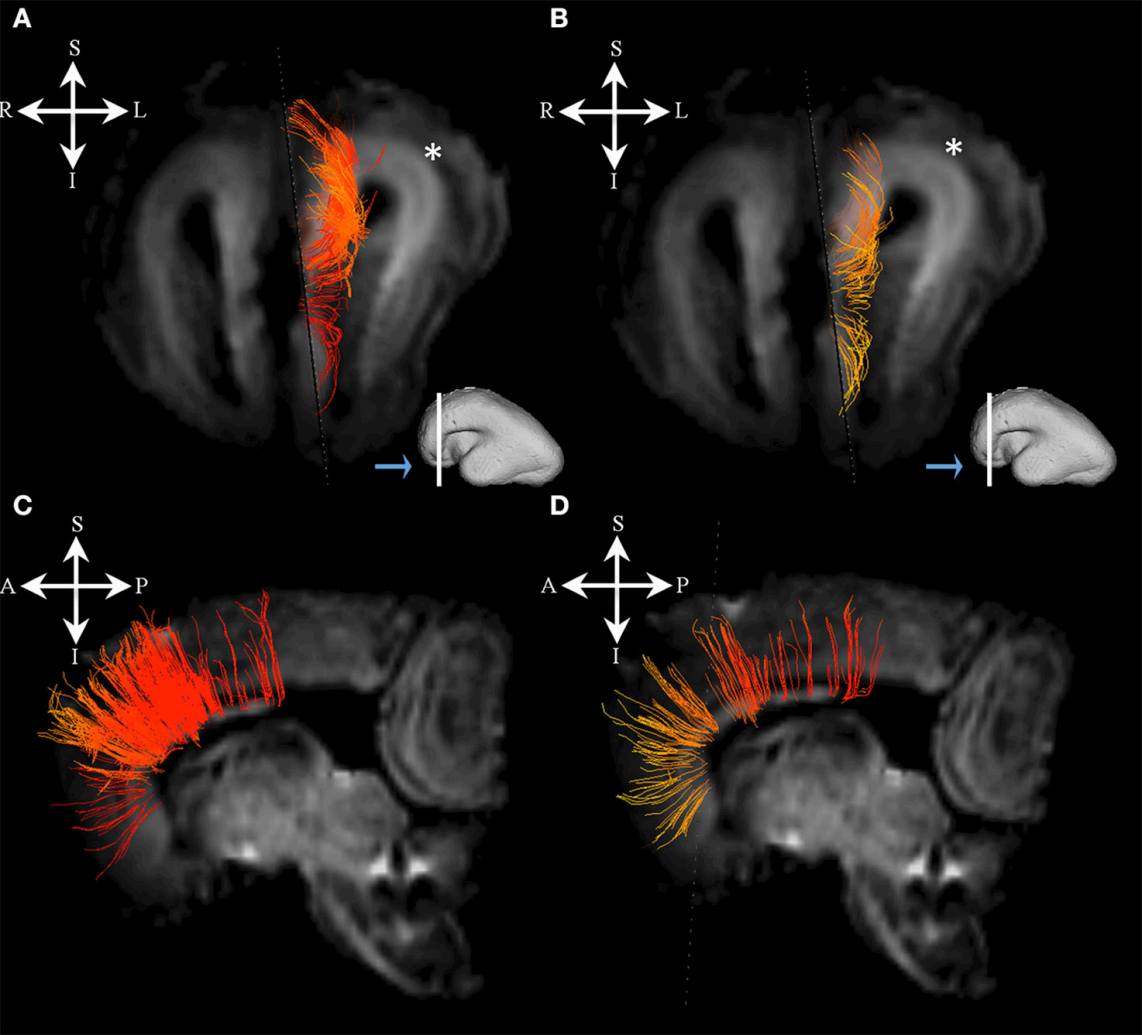
Callosal fibers during mid-fetal period. Reconstruction of callosal fibers in 18 PCW old brain that originate/end in cortical plate **(A,C)** or originate/end in subplate zone **(B,D)**. Coronal and sagittal slices are DWI images of corresponding brains. Adjacent to the each coronal slice **(A,B)** is an illustration of the reference brain surface with the approximate level of the coronal slice (white line), and the view of the slice (anterior or posterior, marked with the blue arrow). Reference orientations [anterior (A), posterior (P), superior (S), inferior (I), left (L), and right (R)] are placed in the left upper corner of each slice. Subplate zone of the frontal lobe is marked with an asterisk.

At this stage callosal fibers were successfully identified. We have identified two sets of callosal fibers: fibers that “origined” in cortical plate (Figures 4A,C), and fibers that “originated” in the subplate zone (in terms of tractography; Figures 4B,D). The subplate zone showed low FA at this stage (Figure 4, asterisk).

#### Association fibers

**Figure 5:**
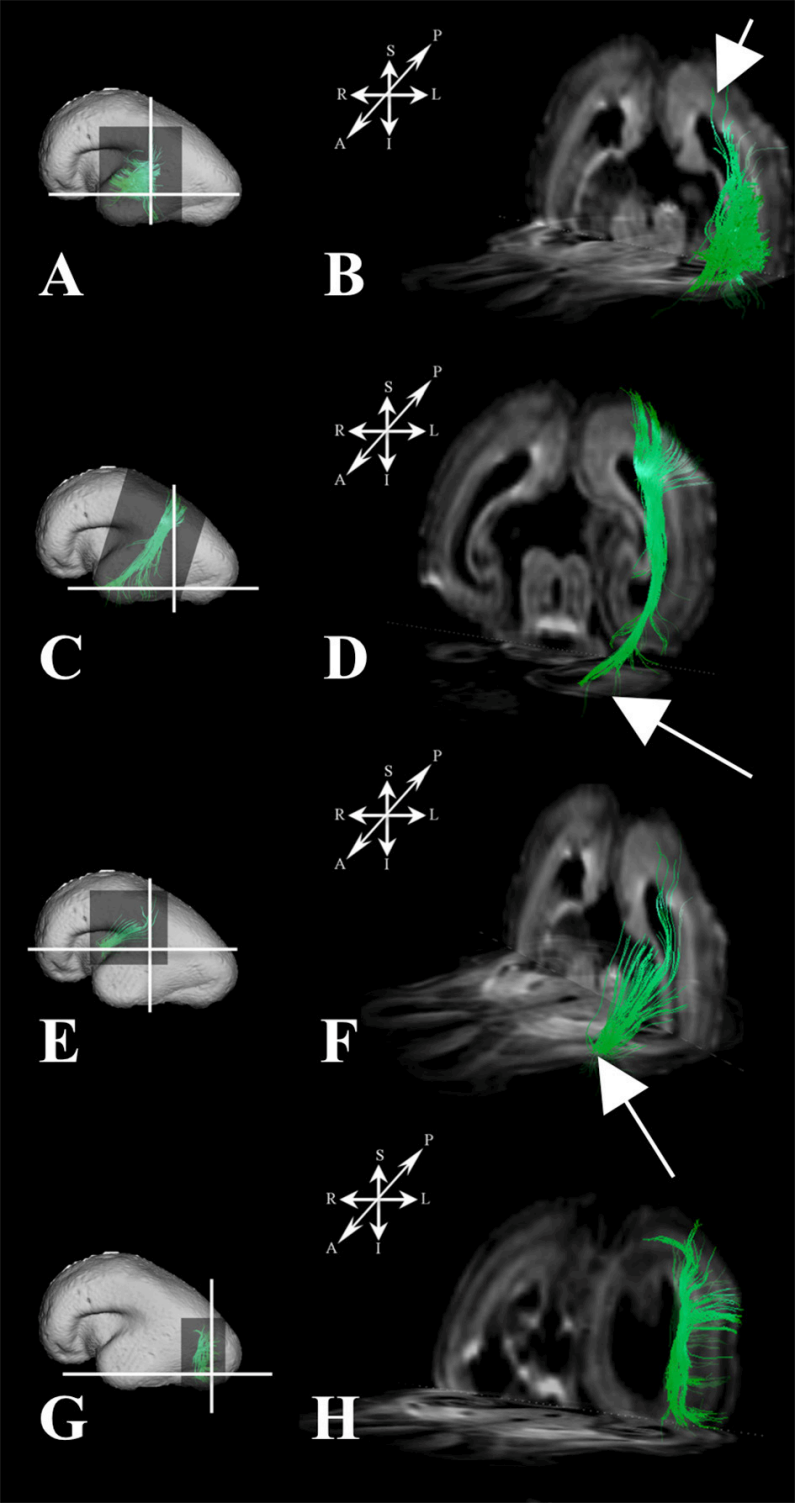
Association fiber pathways during mid-fetal period. Reconstruction of association cortical fiber bundles in formation [middle longitudinal fasciculus **(A,B)**, fiber bundle that connects cortical plate of the superior parietal lobule and temporal pole **(C,D)**, inferior fronto-occipital fasciculus **(E,F)**, and vertical occipital fasciculus **(G,H)**] in 18 PCW old brain. In order to demonstrate position of these fibers in the brain, fibers were reconstructed in 3D and superimposed to the reconstructed surfaces of the similar age brains **(A,C,E,G)** from Zagreb Neuroembryological Collection. White lines in **(A,C,E,G)** show the levels of coronal and axial sections shown in **(B,D,F,H)**. Reference orientations [anterior (A), posterior (P), superior (S), inferior (I), left (L), and right (R)] are placed in the left upper corner of each composite cut. Arrow in **(B)** indicates deep portion of subplate zone, arrow in **(D)** shows temporal pole, arrow in **(F)** indicates external capsule.

During this period we have identified several prospective long cortico-cortical pathways that were still in formation (Figures 5A–H):

1. middle longitudinal fasciculus (Figures 5A,B) that originated (in terms of tractography reconstruction) in the caudal part of the future superior temporal gyrus (Figure 5A) reaching a deep portion of the subplate zone of the inferior parietal lobule (Figure 5B, arrow),
2. a fiber bundle that connected superior parietal lobule with the temporal pole (Figures 5C,D),
3. components of inferior fronto-occipital fasciculus (Figures 5E,F) that passed through the external capsule (Figure 5F, arrow),
4. vertical occipital fasciculus connecting superior and inferior regions of cortical plate of the occipital lobe (Figures 5G,H), and
5. fronto-occipital fasciculus (Figures 1B,H green) that was the most prominent in frontal region.

### Early preterm period (23–28 PCW)

#### Projection fibers

**Figure 6:**
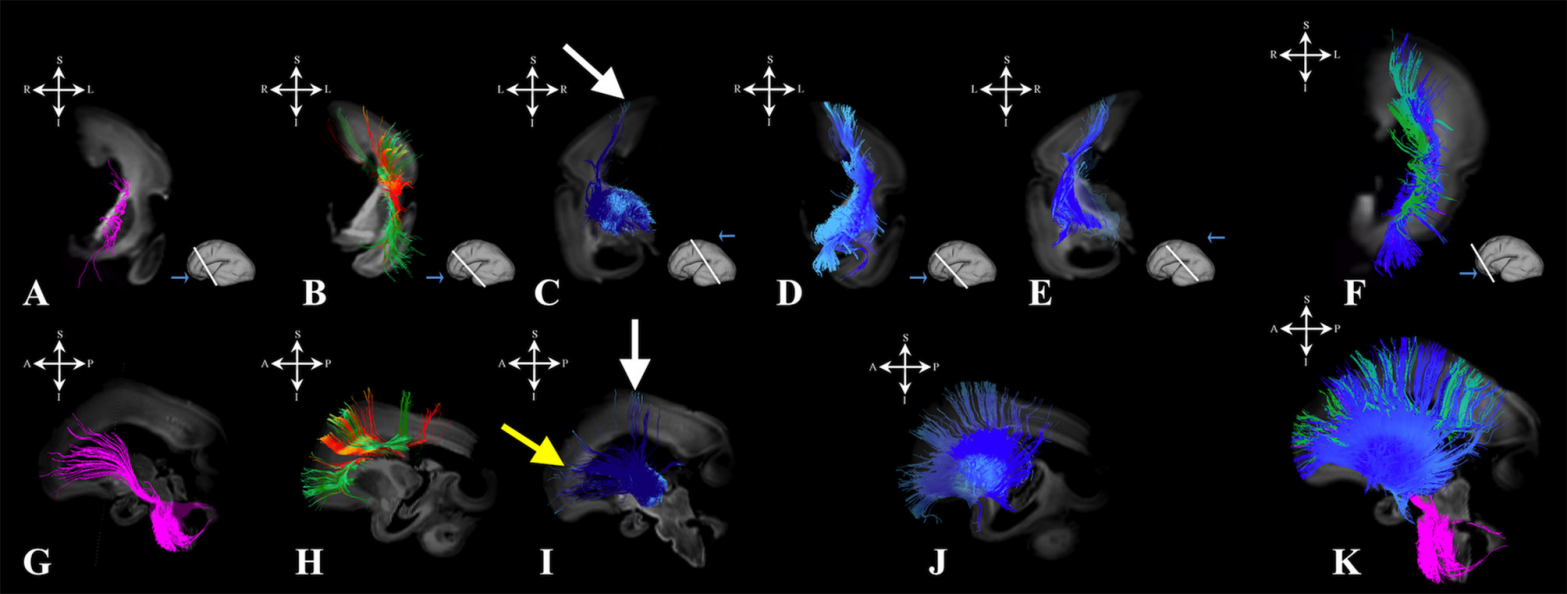
Projection pathways and fasciculus subcallosus during early prenatal period. Reconstruction of projection corticopontine, ponto-cerebellar and fibers related to pons **(A,G)**, fasciculus subcallosus—Muratoff bundle (**B,H** in green), thalamic **(C,I)**, and basal ganglia and basal forebrain fibers conveyed to the external capsule **(D,E,J)** in 24 PCW old brain. Reconstruction of association fronto-occipital fasciculus is shown in **(B,H)** in orange. Composite image of the spatial arrangement of fibers is shown in **(F,K)**. Coronal sections of DWI images are in the upper row, sagittal sections are in the bottom row. Adjacent to the each coronal slice is an illustration of the reference brain surface with the approximate level of the coronal slice (white line), and the view of the slice (anterior or posterior, marked with the blue arrow). Reference orientations [anterior (A), posterior (P), superior (S), inferior (I), left (L), and right (R)] are placed in the left upper corner of each slice. White arrows indicate voluminous portion of thalamocortical fibers reaching the cortical plate in central regions. Yellow arrow in **(I)** indicate thalamocortical fibers that reach the subplate (but not the cortical plate) of the frontal regions.

During the early preterm period, we have seen further increase in volume of pontine, tegmental, and cerebellar fiber pathways (Figures 6A,G). These fibers were seen connecting the cortical plate of the frontal lobe and subcortical structures (Figure 6, note that the majority of fibers connect the cortical plate and subcortical structures). Compared to the previous stage, the thalamo-cortical bundle was more voluminous, especially in central brain regions (Figures 6C,I white arrows). In the frontal regions, the majority of the fibers of the thalamo-cortical system connected the subplate zone in the prefrontal cortex and the thalamus (Figure 6I yellow arrow**)**. During the early preterm phase, fibers originating from the lateral geniculate body were also identified (Figure 2B). These fibers were more numerous compared to the previous stage, but still did not reach the occipital cortex. Furthermore, at this stage, a subcallosal fasciculus—Muratoff bundle (Figures 6B,H,F,K green), and fibers that conveyed to the external capsule from basal ganglia and basal forebrain (Figures 6D–F,J,K) were connecting the cortical plate with subcortical structures.

#### Radial coherence

At this stage we observed the radial streamlines connecting proliferative zones (ventricular and subventricular zone) with the cortical plate (Figure 3B red) or subplate (Figure 3B yellow). The radial streamlines that stretched from proliferative zones to cortical plate were more voluminous than in the previous stage (Figure 3B red), especially in parietal and frontal lobes. However, this period was marked by the abundant radial streamlines originating (in terms of fiber tracking) from the cortical plate that did not reach the proliferative zones (ventricular and subventricular zone) and did not have a well-defined ending structure (Figure 3B green), e.g., they “ended” in the subplate or intermediate zone (not shown).

#### Callosal fibers

Callosal fibers, at this stage, were more abundant but of a similar composition to the callosal fibers described in the previous stage ending/originating in cortical plate or subplate (not shown).

#### Association fibers

**Figure 7:**
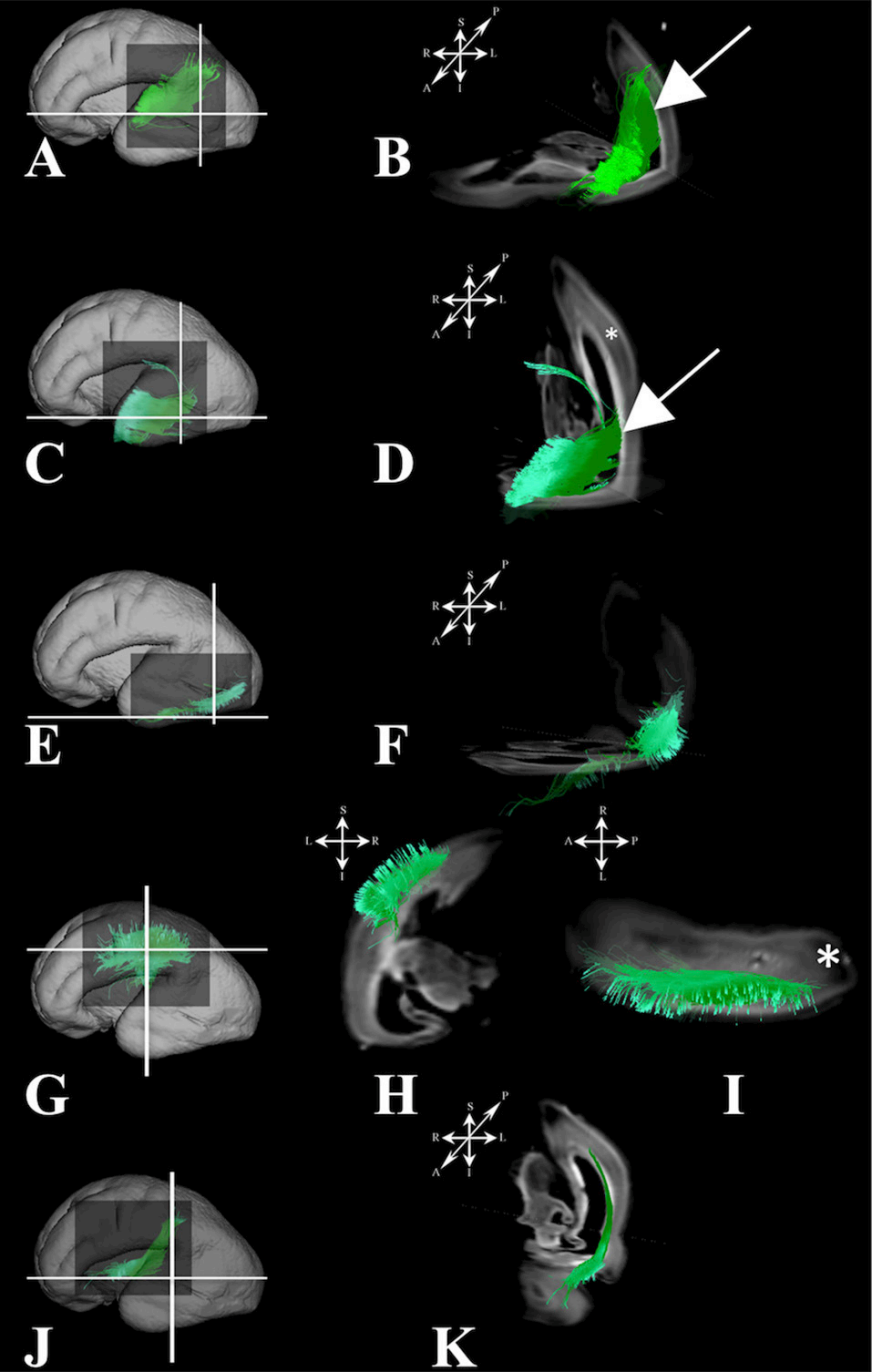
Association fibers during early prenatal period. Reconstruction of association fiber bundles in formation (components of the middle longitudinal fasciculus originating from caudal **(A,B)** or rostral **(C,D)** part of superior temporal gyrus, inferior fronto-occipital fasciculus **(J,K)**, inferior longitudinal fasciculus **(E,F)**, and parts of superior longitudinal fasciculus**(G–I)** in 24 PCW old brain. In order to demonstrate position of these fibers in the brain, these fibers were reconstructed in 3D and superimposed to the reconstructed surfaces of the similar age brains **(A,C,E,G,J)** from Zagreb Neuroembryological Collection. White lines in **(A,C,E,G,J)** show the levels of adjacent coronal and axial sections in **(B,D,F,H,I,K)**. Reference orientations [anterior (A), posterior (P), superior (S), inferior (I), left (L), and right (R)] are placed in the left upper corner of each composite cut. Subplate zone is marked with ^*^. Arrows in **(B**,**D)** indicate “deeper” part of subplate zone.

Association fibers that were identified during mid-fetal period were found to be more voluminous and longer (Figure 7) than in the prior mid-fetal period. At this stage, components of the middle longitudinal fasciculus (Figures 7A–D) were seen to “originate” from the entire superior temporal gyrus. Depending on its anatomical origin (caudal (Figures 7A,B) or rostral (Figures 7C,D) part of the superior temporal gyrus), the middle longitudinal fasciculus traveled in the superior (Figure 7B white arrow) or inferior (Figure 7D white arrow) portion of the deep subplate zone (Figure 7B asterisk) in the parietal lobe. This was the earliest stage at which we could identify components of the interior longitudinal fasciculus (Figures 7E,F). Similarly, at this stage, we also have been able to identify some of the components of the superior longitudinal fasciculus (SLF) (Figures 7G–I). The SLF in formation occupied superficial regions of the dorsolateral subplate zone (Figure 7I asterisk) of the frontal and parietal lobe. Lastly, the inferior fronto-occipital fasciculus was more developed at this stage (Figures 7J,K), i.e., it was longer and could be traced within the frontal and temporal lobes.

### Late preterm period (28–34 PCW)

#### Projection fibers

At this stage all the projection fibers were maximally developed (not shown).

#### Radial coherence

During late preterm period the radial streamlines originating (in terms of tractography) in proliferative zones were more sparse (Figure 3C) compared to the previous periods. Thus, radial coherence of telencephalic wall was less pronounced. In addition, growing cortical efferents forming a U-shape (Figure 3C arrow) predominated.

#### Callosal fibers

**Figure 8:**
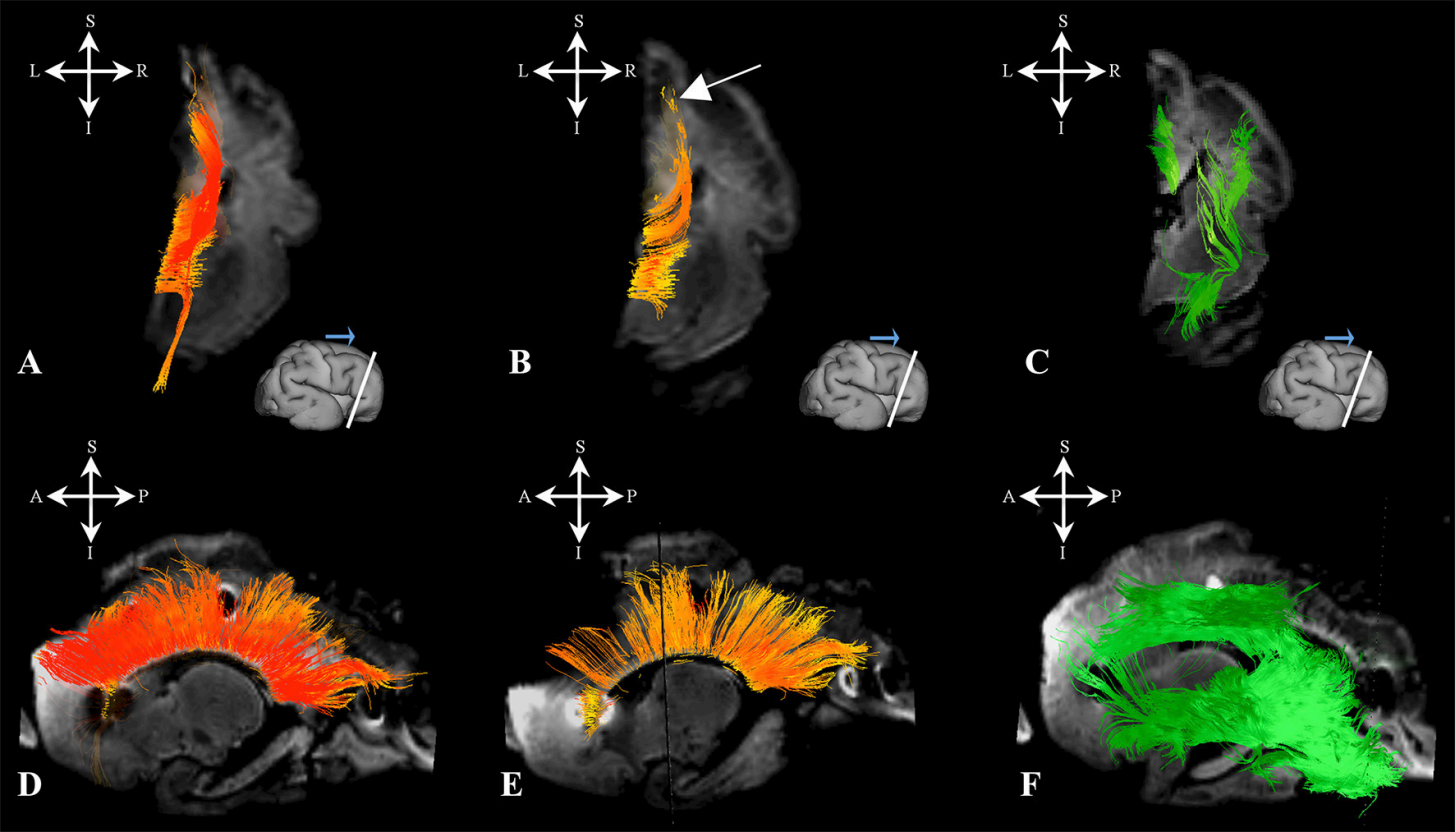
Callosal fibers during late prenatal period. Reconstruction of callosal axons in 29 PCW old brain that originate or end in the cortical plate **(A,D)** or the subplate zone **(B,E)**. Reconstruction of long association fiber bundles in the same brain **(C,F)**. Coronal sections of DWI images are in the upper row, sagittal sections are in the bottom row. Adjacent to the each coronal slice is an illustration of the reference brain surface with the approximate level of the coronal slice (white line), and the view of the slice (anterior or posterior, marked with the blue arrow). Reference orientations [anterior (A), posterior (P), superior (S), inferior (I), left (L), and right (R)] are placed in the left upper corner of each slice. Arrow in **(B)** indicates the fibers that did not end/origin in cortical plate.

During this stage, we have observed an abundant callosal system (Figures 8A,B,D,E). However, a significant portion of callosal fibers did not reach the cortical plate (Figures 8B,E arrow).

#### Association fibers

**Figure 9:**
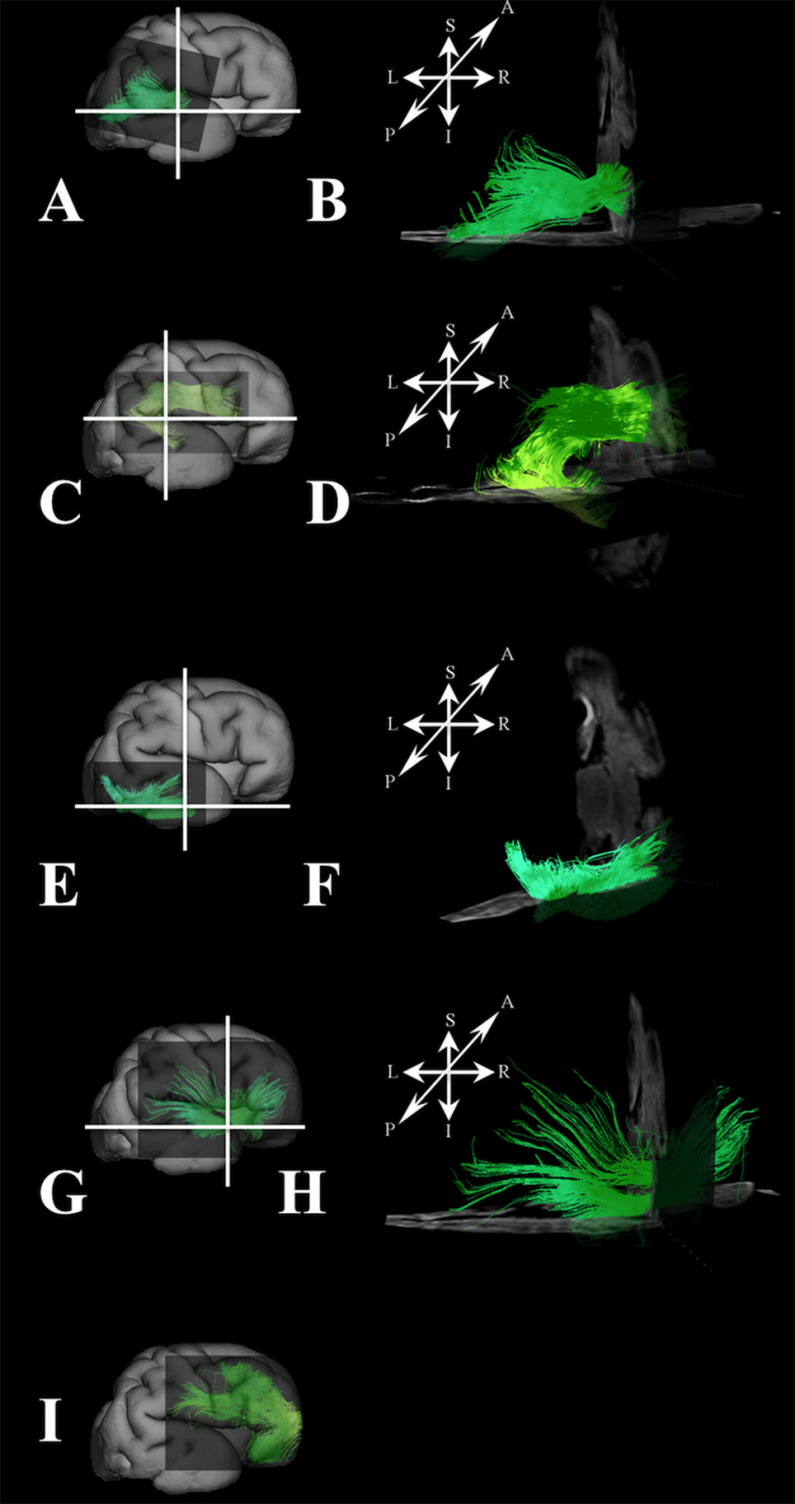
Association fibers during late prenatal period. Reconstruction of growing corticofugal axons in the frontal lobe (I), and formation of long association cortical fiber bundles [middle longitudinal fasciculus **(A,B)**, inferior fronto-occipital fasciculus **(G,H)**, inferor longitudinal fasciculus **(E,F)**, superior longitudinal fasciculus **(C,D)** in 30 PCW old brain]. In order to demonstrate position of these fibers in the brain, these fibers were reconstructed in 3D and superimposed to the reconstructed surfaces of the similar age brains **(A,C,E,G,I)** from Zagreb Neuroembryological Collection. White lines in **(A,C,E,G)** show the levels of adjacent coronal and axial sections in **(B,D,F,H)**. Reference orientations [anterior (A), posterior (P), superior (S), inferior (I), left (L), and right (R)] are placed in the left upper corner of each composite cut.

At this stage, long association fibers predominated in the fetal telencephalon, occupying the majority of its volume. Figures 8C,F are composite images of all the long association fiber bundles that were reconstructed in the brain. These growing long association fiber pathways were mostly composed of the middle longitudinal fasciculus (Figures 9A,B), components of the SLF that were easily identified in the caudal portion of the frontal lobe and the rostral portion of the inferior parietal lobule (Figures 9C,D), the inferior longitudinal fasciculus (Figures 9E,F), and the inferior fronto-occipital fasciculus (Figures 9G,H). All of these fiber pathways were more voluminous compared to the previous stage. Off note, in the rostral parts of the frontal lobe, we could only reconstruct growing corticofugal axons (Figure 9I) and not compact bundles.

### Sagittal arrangement of fibers in periventricular and intermediate zone (18–34 PCW)

**Figure 10:**
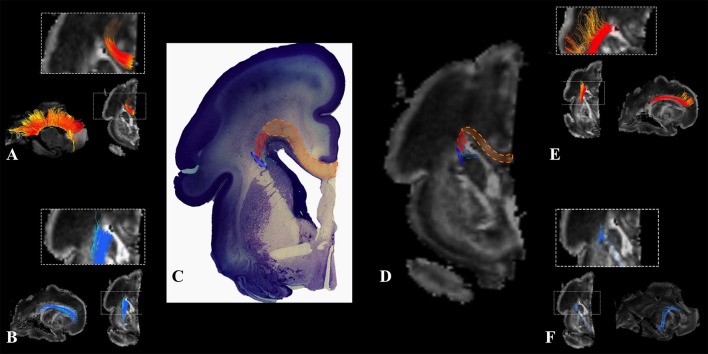
Frontal periventricular fiber system during late preterm period. Reconstruction of fibers that pass through the periventricular fiber rich zone in 30 PCW old brain. Association and callosal fibers are in orange, projection fibers are in blue. Callosal fibers **(A)**, fronto-occipital fasciculus **(E)**, subcallosal fasciculus **(B)**, and corticopontine fibers **(F)**. Nissl stained section **(C)** and corresponding FA slice **(D)** show regions in the periventricular fiber rich zone where fibers of callosum (orange), fronto-occipital fasciculus (red), subcallosal fasciculus (sea green), and corticopontine system (blue) pass.

**Figure 11:**
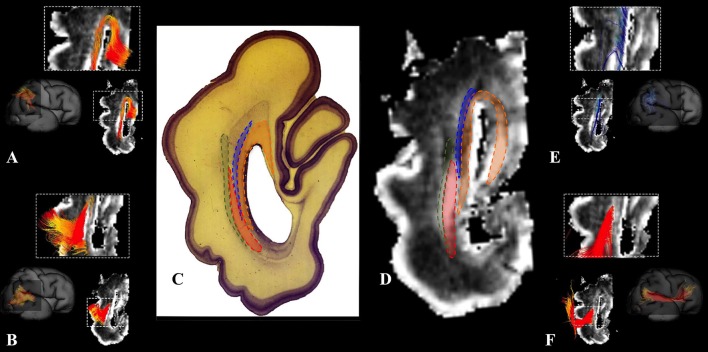
Occipital periventricular fiber system during late preterm period. Reconstruction of fibers that pass through the periventricular fiber rich zone in the occipital lobe in the 30 PCW old brain. Callosal fibers **(A)**, efferent fibers from posterior temporal cortex **(B)**, thalamocortical fibers **(E)**, and inferior fronto-occipital fasciculus **(F)**. Nissl stained section **(C)** and corresponding FA slice **(D)** show regions where fibers of callosum (orange), thalamocortical system (blue), prospective efferent fibers from posterior temporal cortex (red), and inferior fronto-occipital fasciculus (green) pass.

We have identified a sagittal arrangement of fibers during the prenatal human brain development (Figures 10C,D and 11C,D). It can be seen in the mid-fetal (Figure 1F), early (Figure 6), and late preterm periods (Figure 10), that the corpus callosum (Figure 10A), the fasciculus subcallosus (Figure 10B), the fronto-occipital fasciculus (Figure 10E), and the cortico-pontine fibers (Figure 10F) were topologically positioned in the periventricular fiber rich zone (Figures 10C,D). In the occipital lobe, callosal fibers (Figure 11A) and thalamocortical fibers (Figure 11E) were positioned closer to the ventricle, while the prospective association fibers from the posterior temporal cortex (Figure 11B) and inferior fronto-occipital fasciculus (Figure 11F) were positioned more laterally.

## Discussion

In this study, we used an advanced method for fiber pathway reconstruction (HARDI and Q-ball reconstruction in combination with deterministic streamline tractography) that can reliably resolve crossings of fiber pathways in the developing brain. Our results show that in the human fetal brain: (1) massive projection fibers have already developed by 18 PCW while association fibers (with the exception of limbic ones) have only begun to emerge, (2) these pathways show a characteristic laminar distribution and sagittal plane geometry, (3) the corpus callosum and association cortical pathways show protracted prenatal development, and (4) an early prenatal period (24 PCW) is marked by radial coherence (majority of reconstructed streamlines in the telencephalic wall had radial orientation) of the telencephalic wall. These results could be useful for identification of neuronal substrates in children with developmental disabilities that suffered from perinatal brain injuries.

### Maturation of major fiber pathways during midfetal and preterm periods

Although our findings on development of fiber systems in this study are limited to the period of 18 to 34 PCW (when most fiber pathways have already develop their trajectories), our results show some developmental trends in growth of certain corticopetal and corticofugal fiber pathways. First of all, corticopetal and corticofugal fiber pathways connecting the cortex with subcortical structures become more massive and voluminous during the developmental period studied (18 to 30 PCW; Figures 1, 2, 6). This is obvious for thalamocortical fibers (Figures 1C,I, 6C,I, 2), which accumulate in the subplate between 19 and 22 PCW and penetrate the cortical plate around 24 PCW (Figure 6I, arrows; Molliver et al., 1973; Kostovic and Rakic, 1990; Kostovic and Judas, 2002). In addition, early growth of the optic tract and optic radiation, followed by their early myelination (Kinney et al., 1988), suggest that anatomical substrate for visual information processing is among the first ones to mature. A similar developmental trend was observed in corticopetal projection fibers which run from the basal forebrain and tegmentum to the neocortex, projecting though the external capsula (Figures 1E,K, 6D,E,J,K), as well as the medial hypothalamic septal root (Kostović, 1986).

Due to the close spatial relationship between the projection fibers that run from the basal forebrain to the external capsula and the inferior frontooccipital fascicle [IFOF, which develops relatively late (Figures 7J,K)], making a clear distinction between the two became difficult.

Growing corticofugal projection fibers near the cortex are the most difficult to visualize by diffusion tractography (e.g., Takahashi et al., 2010). This is likely due to the fact that fiber fascicules with small diameters enter the voluminous extracellular matrix-rich subplate compartment (Kostovic et al., 2002; Duque et al., 2016; Figure 1, asterisk). We have encountered similar difficulty in visualization of pathways passing through the subplate compartments in early stages (Figure 1A). Thus, our results suggest that corticofugal fibers from the cortical plate neurons pass through the subplate as fascicles with very small volume fractions, which are hard to image. However, upon entering the intermediate zone (future corona radiata), these fascicles most likely form larger fascicles, with larger volume fractions that are easier to image.

Our results are in agreement with our previous findings (Vasung et al., 2011), showing that the periventricular frontooccipital fasciculus (FOF) develops early in life (Figure 1B). In contrast, other, more laterally situated, long association pathways form a part of the centrum semiovale during the mid fetal period, and develop pronanunced trajectory much later (Huang et al., 2006; Kasprian et al., 2008; Song et al., 2015). For example, the SLF in our materials was not clearly visible until 29 PCW. Thus, growth and development of long (lateral) association pathways is delayed for at least 2 months compared to the development of thalamocortical fibers, which is well in agreement with a past histology study (Kostovic and Goldman-Rakic, 1983).

### Organization of major fiber pathways and their relationships with transient fetal zones during midfetal and preterm periods

Consistent with previous studies (Kostovic and Judas, 2002; Huang et al., 2009; Kostovic et al., 2014), we found major fiber pathways in three major transient zones: the periventricular zone (Figure 1: red arrows), the intermediate zone (Figure 1F: iz), and the subplate (Figure 1F: asterisks). Our results show that major projection corticofugal (subcallosal fasciculus, corticopontine) and corticopetal (thalamocortical, basal forebrain) fibers travel across the three zones at 18 PCW. We have found that corticofugal projection pathways show deeper (closer to the ventricles) positioning (e.g., subcallosal fasciculus and corticopontine pathways were situated in the periventricular fiber rich zone, at the level of corticostriatal junction) compared to corticopetal thalamocortical fibers, which run more superficially (toward pia; Figure 1F). This finding is in agreement with previous studies (Vasung et al., 2011).

Another structural component that displayed high FA values was previously described by Huang et al. (2006) and Takahashi et al. (2012). The structure is situated more medially than FOF in the periventricular region, and seems to be related to ganglionic eminence (Huang et al., 2006; Takahashi et al., 2012). The FOF, a corticofugal association pathway (Figure 1F: green, Figure 10E) develops very early and remains close to the ventricle. Therefore, this position makes this bundle especially vulnerable in conditions such as focal periventricular leukomalacia (Volpe, 2012; Kostovic et al., 2014).

Thalamocortical, basal forebrain (Figures 1E,K), and tegmental pathways (Figures 1D,J) are situated away from the ventricle, in a lateral portion of the intermediate zone (Figure 1F: iz-intermediate zone, thalamocortical fibers in blue, tegmental in purple, and fibers from basal forebrain in light blue). A previous histological study showed that early tegmental afferents are composed of fibers with extremely thin calibers (Kostovic and Judas, 2002) that might be difficult to visualize by diffusion MRI tractography. However, it is possible that some tegmental fibers are packed tightly enough for us to be able to detect them in this study. The most massive projection fibers originate in the thalamus (Kostovic and Goldman-Rakic, 1983; Kostovic and Rakic, 1984; Vasung et al., 2010), showing both radial and sagittal plane geometries and run through the capsula interna, crossroads, sagittal strata, and corona radiate (Figures 1C,I, 2A). The external capsule contains sagittally oriented fibers composed of the basal forebrain (i.e., corticopetal fibers), corticostriatal, and association fibers.

### Geometry of major fiber pathways

In terms of geometry, projection fibers were first sagitally oriented (Figure 1, bottom row) in the periventricular or intermediate zones. However, at various points along their trajectory, their direction changed to a radial orientation (Figure 1, upper row) in order to be directed toward the developing subplate compartment of the cerebral wall (Figure 1, asterisks). At 18 PCW, relatively short association fibers (Figure 5) seem to emerge from the cortical plate and invade the deepest portion of the subplate zone (Figure 5, arrows). However, in the FOF, relatively long association fibers (Figure 1F: green, Figure 10E) developed very early, already penetrated into the periventricular zone, and remained close to the ventricle.

Our findings are in accordance with the previous histological studies (Von Monakow, 1905; Kostovic and Goldman-Rakic, 1983; Kostovic, 1986; Kostovic and Rakic, 1990; Kostovic and Judas, 2002; Kostovic and Vasung, 2009). These previous studies have focused on correlation between diffusion MRI and histology (Kostovic and Judas, 2002), and on human fetuses which have showed a specific laminar arrangement and sagittal plane orientation of major fiber systems through the use of diffusion MRI.

This laminar compartmentalization and sagittal plane orientation was present in the youngest period examined in the present study, and can be followed throughout early preterm periods. However, further maturation of these sagittaly oriented pathways show a tendency to transform into a fan-shaped fibers when exiting the periventricular or intermediate zone, suggested by Kostovic et al. (2002). In fact, the fetal white matter is gradually parcellated into segments (Von Monakow, 1905): the prominent corona radiata, centrum semiovale, and gyral white matter. In contrast to regression of glial-neuronal migratory streams (Takahashi et al., 2012; Xu et al., 2014; Wilkinson et al., 2016) and strong radial orientation of embryonic columns in the cortical plate, the radial organization of fiber pathways become more prominent during development. These two processes are closely related. Namely, massive ingrowths of thalamocortical and other fibers (Kostovic and Goldman-Rakic, 1983; Kostovic and Rakic, 1984; Hevner, 2000) cause lamination of the cortical plate (Kostovic and Judas, 2002), and significant changes related to interaction with rapidly developing dendrites (Mrzljak et al., 1988, 1992), which ultimately can be reflected in changes of cortical FA (Mckinstry et al., 2002).

### Growth of callosal system

One of the most interesting developmental findings is related to the growth of callosal fibers. Massive callosal fibers begin to grow between 10 and 11 PCW (Rakic, 1988) and are well-developed by 18 PCW. Thus, it is logical to assume that a high percentage of callosal fibers arrive at the subplate border zone and cortical plate during the subsequent period (Figures 4A,C). However, our results show that a significant proportion of callosal fibers do not reach the subplate and cortical plate during the developmental period analyzed (Figures 4B,D). It is very likely that some of those “unsuccessful fibers” later retract causing a decrease in number of callosal axons seen in the perinatal periods (Lamantia and Rakic, 1994; Innocenti and Price, 2005). In preterm periods, we found a similar distribution of callosal fibers that enter the cortical plate (Figures 8A,D) or “end” in subplate (Figures 8B,E). Our findings are in agreement with studies on non-human primates that indicate protracted development of the corpus callosum (Lamantia and Rakic, 1990).

### Radial coherence

Our study revealed that the presence of radial streamlines stretching between the ventricular zone and the subplate zone, or cortical plate, was the most prominent around 24 PCW (Figure 3, fibers in yellow and red). It is difficult to interpret what is the real structural counterpart of these radial streamlines. It is logical to assume that the radial orientation of radial glia contributes to diffusion anisotropy of the cerebral wall, especially in the early stages. During the early preterm period (around 24 PCW), radial glia are still well-developed in the human cerebral wall (Schmechel and Rakic, 1979; Kostovic et al., 2015; Colombo, 2016), as late born neurons still migrate along radial glia. Therefore, it is likely that this migratory stream, especially in frontal regions (Figure 3B, Fibers in red), contributes to strong diffusion anisotropy (Takahashi et al., 2012; Xu et al., 2014). Even after this period, transforming radial glia (Schmechel and Rakic, 1979; Colombo, 2016) may still contribute to diffusion anisotropy of the cerebral wall. In particular, this might be the case in the frontal lobe where some of those radially oriented streamlines may correspond to some neuronal/glial migration pathways (Xu et al., 2014; Wilkinson et al., 2016). Our results for 30 PCW show that radial fibers became very curved, which correspond to description of Schmechel and Rakic (1979), and became inhomogeneous (Figure 3C). These changes indirectly indicate reorganization of both the subplate and the cortical plate (Kostovic and Judas, 2002; Mckinstry et al., 2002; Takahashi et al., 2012; Kostovic et al., 2014; Xu et al., 2014). In addition, during the late preterm period, some of these radially oriented streamlines, that stretch from cortical plate to subplate or intermediate zone (Figure 3C green), might also correspond to the increase in size of fasciculi and number of corticofugal fibers. However, that remains to be determined by histological methods.

## Significance

Data on fiber pathway development are important for analysis of e.g., hypoxic ischemic lesions in developing preterm brain. As suggested by Staudt et al. (2004), the efficacy of reorganization of fiber tracts after hypoxic-ischemic lesions decreases with increasing gestational age. In addition, it is not known to which extent these fibers [periventricular contingent of motor, early association and sensory pathways and their distal parts (superficially situated axonal fibers)] are affected by focal leukomalacia, and to which extent their injuries lead to rather complex encephalopathy of prematurity (Volpe, 2012). The early establishment of sagittal plane geometry of growing pathways corroborates the “selective radial vulnerability” of different groups of axons in the early preterm as proposed by Kostovic et al. (2014). For example, fibers closer to the ventricle tend to be more injured following the periventricular focal leukomalacia. Late development of long association pathways, confirmed in the present study, may shift a developmental window of vulnerability for these pathways to a late preterm period (Kostovic et al., 2014). It still remains unclear whether fine abnormalities of fiber pathways seen with HARDI may identify altered fiber pathways in other developmental disorders (Travers et al., 2012; Mcfadden and Minshew, 2013). Therefore, we believe that new histological postmortem findings which include compartmental distribution of axonal, cellular and extracellular components in correlation with 3-dimensional MR imaging methods offer a new direction for analyses of disturbed connectivity-related disorders, such as autism and other developmental cognitive disorders (Fischi-Gomez et al., 2015). Lastly, in depth knowledge about the cellular architecture of the human fetal brain (revealed by histology) would be of significant importance for validation of new diffusion modeling techniques employed in premature infants and newborns (e.g., CHARMED-light and NODDI; Kunz et al., 2014).

## Limitations

The largest limitation of our study is the small number of specimens that were obtained from two different sources (Allen Institute and BWH). Thus, we are unfortunately not able to assess to which point inter-individual differences affect our results. The known pitfalls of the fiber reconstruction using HARDI have been reported in recent publication (Maier-Hein et al., 2016). In short, according to Maier-Hein et al., currently available algorithms for fiber pathway reconstruction tend to produce more than 80% of false positive fibers, especially in periventricular regions (Maier-Hein et al., 2016). Although, our algorithms are similar to ones presented in their paper (Maier-Hein et al., 2016), we propose that compared to the adult the different architecture of fetal telencephalon and shorter fiber pathways most likely lead to a smaller number of reconstructed false positive pathways. However, tractography in human fetal brains still needs to be strictly correlated with histologically identified fibers in order to accurately and convincingly see the sequential growth of fibers. This is mostly because, at these periods of development, growing front of specific fibers may be difficult to demonstrate due to the extremely small diameter of fibers (Mori and Zhang, 2006), which are embedded in voluminous extracellular matrix (Kostovic and Rakic, 1990).

## Ethics statement

This study used the digital MR data (obtained with full parental consent in accordance with the Declaration of Helsinki, and approved by Department of Pathology, Brigham and Women's Hospital) and digital histology data from Zagreb Neuroembryological Collection (obtained with full parental consent in accordance with the Declaration of Helsinki, and approved by ethical committee of School of Medicine, University of Zagreb).

## Author contributions

ET collected the postmortem specimens, scanned them with diffusion MRI, and reconstructed tractography fiber pathways. LV analyzed and identified groups of tracts. MR helped with histology analysis and acquisition. IK and ET contributed to interpretation of results. LV, IK, and ET wrote the manuscript.

### Conflict of interest statement

The authors declare that the research was conducted in the absence of any commercial or financial relationships that could be construed as a potential conflict of interest.

## Supplementary material

Supplementary Figure 1 **Example of the semi-automatic segmentation of the fetal brain during mid-fetal period**. Fractional anisotropy **(A,B)** and dwi axial slices **(C,D)** used for segmentation of the cortical plate (red), proliferative zones (light blue), subplate zone (purple), intermediate zone (orange), thalamus (dark blue), corpus callosum (yellow), caudate nucleus (green), and ganglionic eminence (pink).
