# Supplementary figures and images for: Spatiotemporal Relationship of Brain Pathways during Human Fetal Development Using High-Angular Resolution Diffusion MR Imaging and Histology
Source: Front Neurosci. 2017 Jul 11;11:348. doi: 10.3389/fnins.2017.00348 (PMC5504538; doi:10.3389/fnins.2017.00348)

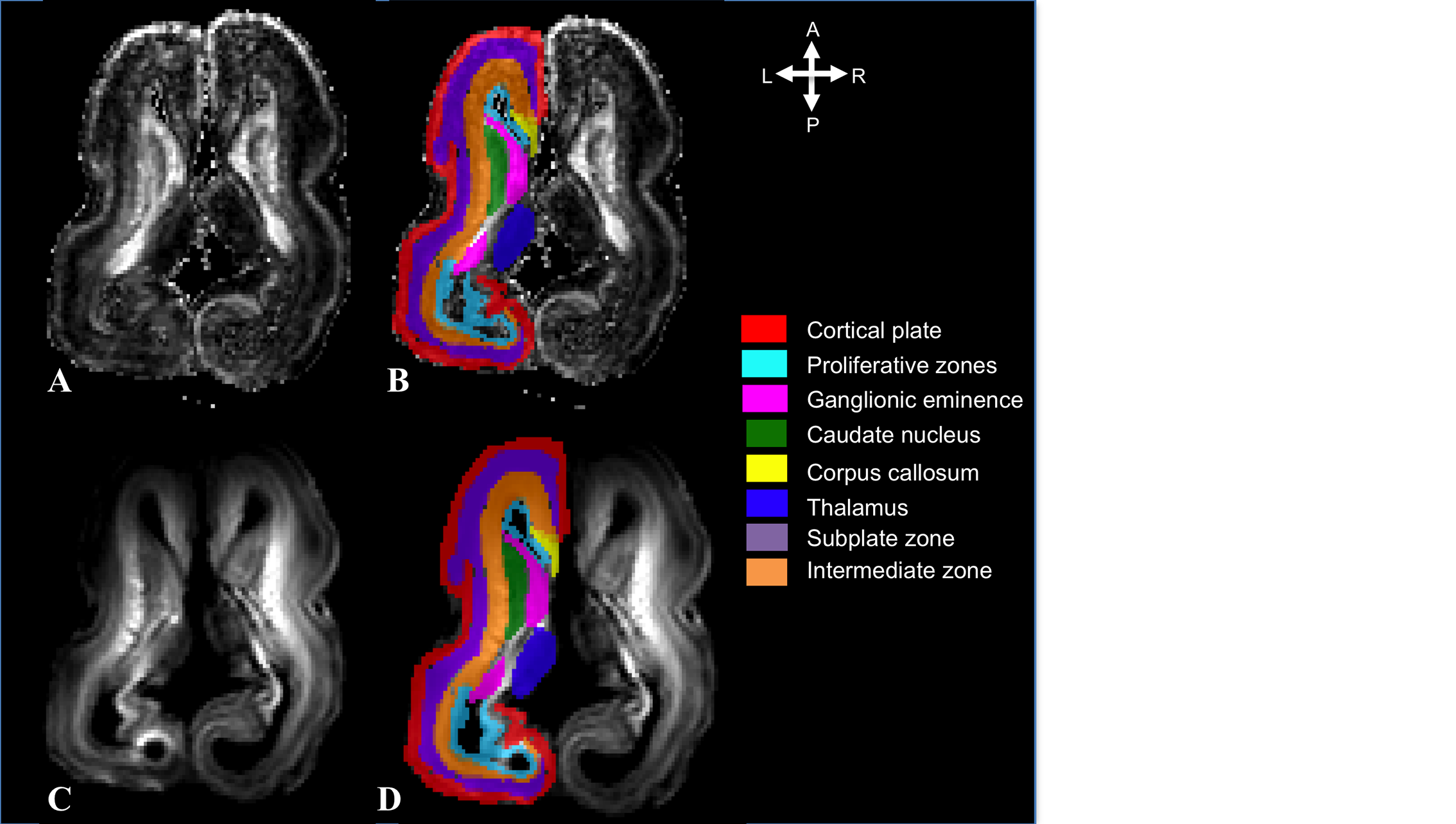

Supplement: Supplementary Figure 1 — Example of the semi-automatic segmentation of the fetal brain during mid-fetal period. Fractional anisotropy (A,B) and dwi axial slices (C,D) used for segmentation of the cortical plate (red), proliferative zones (light blue), subplate zone (purple), intermediate zone (orange), thalamus (dark blue), corpus callosum (yellow), caudate nucleus (green), and ganglionic eminence (pink). [file Image1.TIF]
